# Updated trends in the outcomes of out‐of‐hospital cardiac arrest from 2017–2021: Prior to and during the coronavirus disease (COVID‐19) pandemic

**DOI:** 10.1002/emp2.13070

**Published:** 2023-11-27

**Authors:** Cheng‐Yi Fan, Chih‐Wei Sung, Ching‐Yu Chen, Chi‐Hsin Chen, Likwang Chen, Yun‐Chang Chen, Jiun‐Wei Chen, Wen‑Chu Chiang, Chien‐Hua Huang, Edward Pei‐Chuan Huang

**Affiliations:** ^1^ Department of Emergency Medicine National Taiwan University Hospital Hsin‐Chu Branch Hsinchu Taiwan; ^2^ Department of Emergency Medicine College of Medicine National Taiwan University Taipei Taiwan; ^3^ Department of Emergency Medicine National Taiwan University Hospital Yun‐Lin Branch Douliu Taiwan; ^4^ Institute of Population Health Sciences National Health Research Institutes Miaoli Taiwan; ^5^ Department of Emergency Medicine National Taiwan University Hospital Taipei Taiwan

**Keywords:** cardiopulmonary resuscitation, coronavirus disease 2019, outcomes, out‐of‐hospital cardiac arrest, trend

## Abstract

**Objective:**

This study aims to describe out‐of‐hospital cardiac arrest (OHCA) characteristics and trends before and during the coronavirus disease‐2019 (COVID‐19) pandemic in Taiwan.

**Methods:**

We conducted a retrospective cohort study using a 5‐year interrupted time series analysis. Eligible adults with non‐traumatic OHCAs from January 2017 to December 2021 in 3 hospitals (university medical center, urban second‐tier hospital, and rural second‐tier hospital) were retrospectively enrolled. Variables were extracted from the emergency medical service reports and medical records. The years 2020 and 2021 were defined as the COVID‐19 pandemic period. Outcomes included survival to admission after a sustained return of spontaneous circulation, survival to hospital discharge, and good neurological outcomes (cerebral performance category score 1 or 2).

**Results:**

We analyzed 2819 OHCA, including 1227 from a university medical center, 617 from an urban second‐tier hospital, and 975 from a rural second‐tier hospital. The mean age was 71 years old, and 60% of patients were males. During the COVID‐19 pandemic period, video‐assisted endotracheal tube intubation replaced the traditional direct laryngoscopy intubation. The trends of outcomes in the pre‐pandemic and pandemic periods varied among different hospitals. Compared with the pre‐pandemic period, the outcomes at the university medical center during the COVID‐19 pandemic were significantly poorer in several respects. The survival rate on admission dropped from 44.6% to 39.4% (*P* = 0.037), and the survival rate to hospital discharge fell from 17.5% to 14.9% (*P* = 0.042). Additionally, there was a notable decrease in patients' good neurological outcomes, declining from 13.2% to 9.7% (*P* = 0.048). In contrast, the outcomes in urban and rural second‐tier hospitals during the COVID‐19 pandemic did not significantly differ from those in the pre‐pandemic period.

**Conclusions:**

COVID‐19 may alter some resuscitation management in OHCAs. There were no overall significant differences in outcomes before and during COVID‐19 pandemic, but there were significant differences in outcomes when stratified by hospital types.

## 1 INTRODUCTION

### 1.1 Background

To rescue patients with out‐of‐hospital cardiac arrest (OHCA) is challenging for the emergency department (ED). The incidence of OHCA is approximately 84 cases per 100,000 people annually and varies geographically.^1^ For decades, the rate of survival to hospital discharge after the return of spontaneous circulation (ROSC) remains low, ranging from 6.4% to 20.4% (mean, 12%).^1^, ^2^ It was also reported that approximately 8% of survivors of OHCA are discharged with good neurological recovery.^3^

On December 2019, the coronavirus disease 2019 (COVID‐19) pandemic spread worldwide.^4^ COVID‐19 directly worsens the outcomes of OHCA survivors and indirectly influences the lockdown and disruption of health care systems.^5^, ^6^ However, how COVID‐19 influences the OHCA outcomes remains unknown.

### 1.2 Importance

The mortality rate after an OHCA was higher in some European countries ^7^, ^8^, ^9^ and the United States,^10^ in the years before the COVID‐19 pandemic; however, no differences in outcomes were found in other European and American cities.^11^, ^12^ The effect of COVID‐19 on outcomes remained inconsistent in Western countries. In Australia, the rate of survival to hospital discharge decreased in non‐emergency medical services (EMS)‐witnessed OHCA populations,^13^ but remained unchanged in EMS‐witnessed OHCA.^14^ Worse outcomes were noted during the pandemic period in Singapore^15^ and Korea.^16^ However, the patient's enrollment duration of COVID‐19 in previous studies ranged narrowly, most simply from January 2020 to June 2020.^7^, ^9^, ^10^, ^12^, ^15^, ^16^ Little information was known about the effect of COVID‐19 on outcomes from June 2020 through December 2021 in Taiwan.

The early delivery of high‐quality cardiopulmonary resuscitation (CPR) and rapid defibrillation improved patients’ outcomes after an OHCA.^17^, ^18^ During the pandemic, for rescuer safety, the World Health Organization suggested that rescuers wear personal protective equipment during CPR.^19^ During the COVID‐19 pandemic, the time required for donning personal protective equipment might decrease the quality of resuscitation, which may cause poor OHCA outcomes. The COVID‐19 pandemic may lead to poorer outcomes for OHCA due to the increased time required for health care professionals to don personal protective equipment, potentially impacting the quality of resuscitation efforts.^20^ The capacity for resuscitation, encompassing factors, such as airway management, CPR duration, and facility availability, could vary among hospitals of different levels.^6^, ^21^ The knowledge gap pertains to this variation in resuscitation capacity across different hospital levels, which may significantly influence OHCA outcomes.

### 1.3 Goals of this investigation

To bridge the research gap, we sought to study current trends in OHCA outcomes. We assessed differences in OHCA before and during the COVID‐19 pandemic in Taiwan. Furthermore, we compared the OHCA outcomes among a university medical center, an urban second‐tier community hospital, and a rural second‐tier community hospital.

## 2 MATERIALS AND METHODS

### 2.1 Study design, participants, and setting

This study conformed to the recommendations by the STROBE statement and was approved by the institutional review board of the NTUH Hsin‐Chu Branch (code: 110‐152‐E) and research ethics committee of National Health Research Institutes (code: EC1101217‐E). Written informed consent was waived.

We conducted a retrospective cohort study using a 5‐year interrupted time series analysis to determine the effects of COVID‐19 pandemic on OHCA in Taiwan. The study was conducted at the National Taiwan University Hospital (NTUH) and its affiliated hospitals. NTUH (the university medical center), with approximately 200 ICU beds, is located in Taipei City. Its 2 affiliated hospitals are a second‐tier urban hospital in Hsinchu City (the urban second‐tier hospital) and rural hospital in Yunlin County (the rural second‐tier hospital), respectively. The available ICU capacities for post‐resuscitation care are 50 beds and 18 beds, respectively. All ICUs in 3 hospitals can provide post‐resuscitation care, including extracorporeal membrane oxygenation and targeted temperature management (Table S1). The annual calls of EMS system in 3 hospitals are approximately 120,000 (the university medical center), 40,000 (the urban second‐tier hospital), and 30,000 (the rural second‐tier hospital), respectively. The Taipei EMS, Hsinchu EMS, and Yunlin EMS contain approximately 92, 64, and 39 ambulance units, respectively. OHCA rescuers in Taipei EMS include 2 ambulance teams with at least 1 emergency medical technician‐paramedic (EMTP). OHCA rescuers in Hsinchu and Yunlin EMS include 1 ambulance team but EMTPs are not always available at any time. Epinephrine administration, endotracheal tube intubation, and intraosseous catheter are generally performed by EMTPs (Table S1).

OHCA cases were proactively identified via a comprehensive review of hospital charts. Three ED physicians conducted the processes of inclusion and exclusion in the monthly study meeting. We included OHCA from January 2017 to December 2021 matching the following inclusion criteria: (1) adults >20 years old; (2) non‐traumatic OHCA; and (3) ED visits with receiving resuscitation and/or post‐cardiac arrest care. We excluded: (1) hospital transfer after initial management at the ED; (2) missed or restricted medical record accessibility; (3) ambiguous record in outcomes; and (4) obvious death. We defined obvious death for patients with decay, rigor, charred black corpse, headless, or visceral overflow.

### 2.2 Data collection and variables

After the eligibility process was completed, 2 independent research assistants collected demographics and covariates from documented and e‐medical charts using the Research Electronic Data Capture (REDCap) system. REDCap is a web‐based software platform designed to support data capture for research studies.^22^ The medical chart review process adhered to rigorous steps to minimize information biases. This study was started in January 2022 and lasted 1 year (till December 2022). The principal investigator held study meetings regularly. In the monthly study meeting, the principal investigator and the supervision investigators assessed the data quality and discussed inconsistencies in the data collection between the research assistants.

Age, sex, and body mass index (BMI) were collected from the e‐medical chart system, including the resuscitation notes. Covariates included prehospital ROSC, outdoor CPR, immediate “do not resuscitate” (DNR) at ED, termination of resuscitation (TOR), airway management, cardiac rhythm, CPR duration, and ED charges. Prehospital ROSC was any ROSC on the ambulance from the scene of the OHCA to the hospital. Outdoor CPR was defined as an OHCA patient who received CPR in an isolated room outside the emergency room during the COVID‐19 pandemic. Patients with immediate DNR did not receive any CPR in the ED. TOR were defined as a decision when OHCA patient's families stopped the on‐going CPR in the ED. Video‐assisted intubation included the Glidescope, C‐MAC, and Pentax AWS laryngoscopes. The supraglottic airway refers to the laryngeal mask airway. Patients with any shockable rhythms were defined as those with any pulseless ventricular tachycardia or ventricular fibrillation during CPR and those who received defibrillation. The CPR duration began and ended when the patient entered the resuscitation room and achieved ROSC or were declared dead, respectively.

**The Bottom Line**

COVID‐19 changed out‐of‐hospital cardiac arrest (OHCA) management in Taiwan, and those changes have persisted. The outcome remains similar as pre‐COVID.

### 2.3 Outcomes

The outcomes included sustained ROSC to admission, survival to hospital discharge, and good neurological outcome. Sustained ROSC to admission were defined as patients who eventually achieved sustained ROSC after CPR in the ED and were admitted to an ICU for post‐cardiac arrest care. The neurological outcome was evaluated at hospital discharge by independent intensivists who cared for the patients but did not participate in the study. Glasgow–Pittsburgh Cerebral Performance Category (CPC) scores were used for the quantitative assessment of neurological outcomes. CPC scores of 1 or 2 indicated good neurological outcomes.^23^

### 2.4 Statistical analysis

OHCA patients from January 1, 2017, to December 31, 2019, were classified into the non‐pandemic group, whereas those from January 1, 2020, to December 31, 2021, were in the pandemic group. A statistical analysis was performed using SPSS 26 (International Business Machines Corporation, IBM), and a 2‐sided *P* value of <0.05 was statistically significant. Normality of variables was determined using the Shapiro–Wilk test.^24^ Continuous variables with a normal distribution, presented as mean ± SD, were compared using Student *t* test or analysis of variance, where applicable. Discrete variables, presented as a number (percentage), were compared by χ^2^ test.

To evaluate the potential effects of the COVID‐19 pandemic on resuscitation capacity and decision‐making, we compared 2 distinct groups, representative of the non‐pandemic and pandemic periods, using the Student *t* test for continuous variables and the χ^2^ test for discrete variables. Additionally, to examine the influence of the hospital level on OHCA outcomes, comparisons between the non‐pandemic and pandemic periods were conducted within each individual hospital.

To compare the OHCA characteristics among the enrolled period (from 2017 to 2021), the Bonferroni correction post hoc test was applied to the multiple‐comparison correction. Additionally, multivariable logistic regression was performed to evaluate the association of each independent variable on outcomes. Adjusted odds ratio (aOR) with 95% confidence interval (CI) was used. The significant level in the Bonferroni correction was set to a *P* value of <0.005 (the desired significant level divided by the number of hypotheses, that is, (0.05/$C_{2}^{5})\;$= 0.005).

## 3. RESULTS

3

### 3.1 Study flow of patient enrollment

**FIGURE 1:**
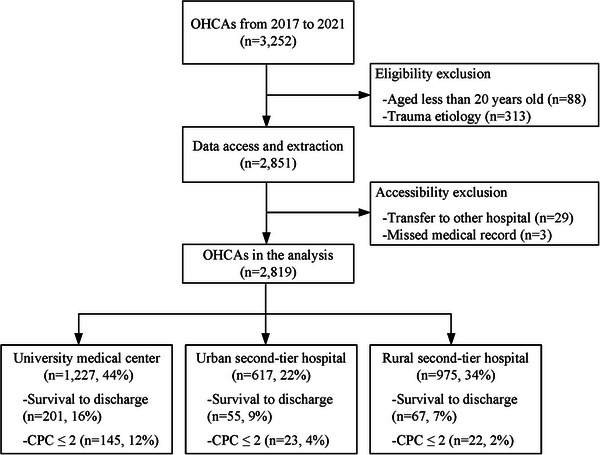
The enrollment of the eligible OHCA patients. OHCA, out‐of‐hospital cardiac arrest.

Figure 1 demonstrates the study flow in enrollment. A total of 3252 documented patients with OHCA were screened initially. Based on the inclusion criteria, 88 pediatric patients and 313 patients with traumatic etiologies (Figure S1) were not included in further data access and extraction. Following the exclusion criteria, 29 patients who visited our ED, received CPR, but transferred to another hospital for post‐cardiac arrest care were excluded because their outcomes were not available. The medical records of 3 patients were missing, therefore, their data were excluded. Finally, 2819 patients were eligible and analyzed. The university medical center had 1227 (43.5%) patients, the urban second‐tier hospital had 617 (21.9%) patients, and the rural second‐tier hospital had 975 (34.6%) patients. In the university medical center, 201 (16%) patients survived hospital discharge and 145 (12%) patients whose CPC scores were less than 2. In the urban second‐tier hospital, 55 (9%) patients survived, and 23 (4%) patients had good neurological outcomes. In the rural second‐tier hospital, the rates of survival and good neurological outcomes were 7% and 2%, respectively.

### 3.2 COVID‐19 effects on resuscitation events and outcomes

Generally, by year, the number of OHCAs ranged from 498 to 619 (approximately 270 per 100,000 ED visits). The mean age trend in OHCA patients remained stable at 71 years old. Most OHCA cases were in males. Approximately 10% of patients arrived in the ED but did not receive any CPR due to their family's decisions. A significant decrease in the termination of CPR in the ED was found. Approximately 25% of patients terminated ongoing CPR in 2017; this number significantly decreased to 16% in 2021 (*P* = 0.001) (Table S2). Table 1 shows the comparison of demographics, resuscitation events, and OHCA outcome between non‐pandemic and pandemic groups. In the pandemic group, patients who received outdoor CPR significantly increased (0% vs. 12.2%, *P* < 0.001). The use of the supraglottic airway significantly increased (0.6% vs. 4.3%, *P* < 0.001), as did video‐assisted endotracheal tube intubations (6.4% vs. 45.3%, *P* < 0.001). Accordingly, traditional direct laryngoscopy dramatically decreased (54.3% vs. 19.4%, *P* < 0.001). The outcomes did not differ between groups.

**TABLE 1:** Comparisons of demographics, resuscitation events, and out‐of‐hospital cardiac arrest outcome between non‐pandemic and pandemic period.

	Non‐pandemic period (*n* = 1605)	Pandemic period (*n* = 1214)	*P*
Age, years	71.3 ± 16.1	70.5 ± 15.7	0.181
Sex, males	969 (60.4)	747 (61.5)	0.533
BMI	22.9 ± 5.0	23.3 ± 5.1	0.076
Hospital			<0.001
University medical center	702 (43.7)	525 (43.2)	
Urban second‐tier hospital	308 (19.2)	309 (25.5)	
Rural second‐tier hospital	595 (37.1)	380 (31.3)	
Prehospital ROSC	140 (8.7)	83 (6.8)	0.066
Outdoor‐CPR	0 (0.0)	148 (12.2)	<0.001
Immediate DNR at ED	225 (14.0)	132 (10.9)	0.013
TOR at ED	362 (22.6)	199 (16.4)	<0.001
Airway management			
Direct laryngoscopy	871 (54.3)	235 (19.4)	<0.001
Video‐assisted	102 (6.4)	550 (45.3)	<0.001
SGA	9 (0.6)	52 (4.3)	<0.001
Tracheostomy	9 (0.6)	18 (1.5)	0.013
Any shockable rhythm	186 (11.6)	175 (14.4)	0.026
CPR duration	20.1 ± 15.2	21.0 ± 14.0	0.182
Sustained ROSC to admission	545 (34.0)	385 (31.7)	0.210
Survival to hospital discharge	189 (11.8)	134 (11.0)	0.543
Good neurological outcome	119 (7.4)	71 (5.8)	0.101

*Note*: Data were presented as mean ± SD or number (percent).

Abbreviations: BMI, body mass index; CPR, cardiopulmonary resuscitation; DNR, do not resuscitate; ED, emergency department; ROSC, return of spontaneous circulation; SGA, supraglottic airway; TOR, termination of resuscitation.

Facing the potential threat of COVID‐19 infection, outdoor CPR was widely applied to OHCA patients. Two groups based on different CPR places were compared (Table S3). A total of 148 (5.3%) of patients received outdoor CPR during the pandemic period. Patients who received outdoor CPR had lower rates of immediate withdrawal, termination of ongoing CPR, and use of direct laryngoscopy. A significantly higher rate of video‐assisted endotracheal tube intubation was noted. In sustained ROSC to admission, a borderline significant decreased outcome was noted in the outdoor group (33.4% vs. 25.7%, *P* = 0.05), whereas the outcome of survival to hospital discharge and good neurological outcomes did not differ between 2 groups (11.6% vs. 8.1%, *P* = 0.19 in survival to hospital discharge; 6.9% vs. 3.4%, *P* = 0.09 in good neurological outcome).

### 3.3 Hospital differences between non‐pandemic and pandemic period on resuscitation events and outcomes

Table 2 demonstrates the comparisons of OHCA in demographics, resuscitation events, and outcomes between non‐pandemic and pandemic period among 3 study hospitals. During the pandemic period, the rate of prehospital ROSC trendily decreased (19.5% to 15.8%, *P* = 0.094, in the university medical center; 19.8% to 15.5%, *P* = 0.164, in the urban second‐tier hospital; 30.6% to 22.4%, *P* = 0.005, in the rural second‐tier hospital). The rate of TOR significantly decreased in 3 hospitals (21.1% to 13.3%, *P* < 0.001, in the university medical center; 32.8% to 24.3%, *P* = 0.019, in the urban second‐tier hospital; 19.0% to 14.2%, *P* = 0.043, in the rural second‐tier hospital). In airway management, intubation by direct laryngoscopy was gradually replaced by video‐assisted intubation in 3 hospitals. More than 40% of intubations were video‐assisted intubation during pandemic period. CPR duration significantly increased in the university medical center (19.2 to 21.4, in minutes, *P* = 0.011), trendily increased in the urban second‐tier hospital (18.8 to 19.8, in minutes), whereas it remained stable in the rural second‐tier hospital (22.2 to 21.3, in minutes). No significant differences in age and sex were found in the 3 hospitals between the 2 periods.

**TABLE 2:** Comparisons of out‐of‐hospital cardiac arrests in demographics, resuscitation events, and outcomes between non‐pandemic and pandemic period between the 3 study hospitals.

	University medical center			Urban second‐tier hospital			Rural second‐tier hospital		
	Non‐pandemic period (*n* = 702)	Pandemic period (*n* = 525)	*P*	Non‐pandemic period (*n* = 308)	Pandemic period (*n* = 309)	*P*	Non‐pandemic period (*n* = 702)	Pandemic period (*n* = 525)	*P*
Age, years	69.8 ± 16.5	68.9 ± 15.6	0.345	71.9 ± 16.6	72.4 ± 16.1	0.723	72.8 ± 15.3	71.2 ± 15.2	0.107
Males	444 (63.2)	349 (66.5)	0.242	186 (60.4)	173 (56.0)	0.268	339 (57.0)	225 (59.2)	0.491
BMI	23.7 ± 4.7	23.3 ± 4.9	0.295	22.4 ± 4.9	23.5 ± 5.6	0.047	22.2 ± 5.2	23.2 ± 4.9	0.012
Prehospital ROSC	137 (19.5)	83 (15.8)	0.094	61 (19.8)	48 (15.5)	0.164	182 (30.6)	85 (22.4)	0.005
Immediate DNR at ED	27 (3.8)	16 (3.0)	0.452	51 (16.6)	46 (14.9)	0.568	147 (24.7)	70 (18.4)	0.021
TOR at ED	148 (21.1)	70 (13.3)	<0.001	101 (32.8)	75 (24.3)	0.019	113 (19.0)	54 (14.2)	0.043
Airway management									
Direct laryngoscopy	358 (51.0)	44 (8.4)	<0.001	160 (51.9)	82 (26.5)	<0.001	353 (59.3)	109 (28.7)	<0.001
Video‐assisted	69 (9.8)	285 (54.3)	<0.001	15 (4.9)	115 (37.2)	<0.001	18 (3.0)	150 (39.5)	<0.001
SGA	1 (0.1)	40 (7.6)	<0.001	0 (0.0)	1 (0.3)	1.000	8 (1.3)	11 (2.9)	0.088
Tracheostomy	4 (0.6)	13 (2.5)	0.006	4 (1.3)	2 (0.6)	0.450	1 (0.2)	3 (0.8)	0.305
Any shockable rhythm	82 (11.7)	76 (14.5)	0.148	26 (8.4)	26 (8.4)	0.990	78 (13.1)	73 (19.2)	0.026
CPR duration	19.2 ± 14.1	21.4 ± 13.2	0.011	18.8 ± 14.8	19.8 ± 14.3	0.422	22.2 ± 16.6	21.3 ± 14.8	0.484
Sustained ROSC to admission	313 (44.6)	207 (39.4)	0.037	81 (26.3)	80 (25.9)	0.908	151 (25.4)	98 (25.8)	0.886
Survival to hospital discharge	123 (17.5)	78 (14.9)	0.042	27 (8.8)	28 (9.1)	0.898	39 (6.6)	28 (7.4)	0.624
Good neurological outcome	94 (13.2)	51 (9.7)	0.048	12 (3.9)	11 (3.6)	0.826	13 (2.2)	9 (2.4)	0.851

*Note*: Data were presented as mean ± SD or number (percent).

Abbreviations: BMI, body mass index; CPR, cardiopulmonary resuscitation; DNR, do not resuscitate; ED, emergency department; ROSC, return of spontaneous circulation; SGA, supraglottic airway; TOR, termination of resuscitation.

As compared with the outcomes in the pre‐COVID‐19 period, the university medical center had significantly poorer outcomes after the COVID‐19 outbreak (ROSC to admission 44.6% vs. 39.4%, survival to hospital discharge 17.5% vs. 14.9%, and good neurological outcome 13.2% vs. 9.7%). In contrast, for OHCA outcomes, no significant differences were found in the urban second‐tier hospital or the rural second‐tier hospital.

### 3.4 Outcomes comparison by years (from 2017 to 2021) among the hospitals

**FIGURE 2:**
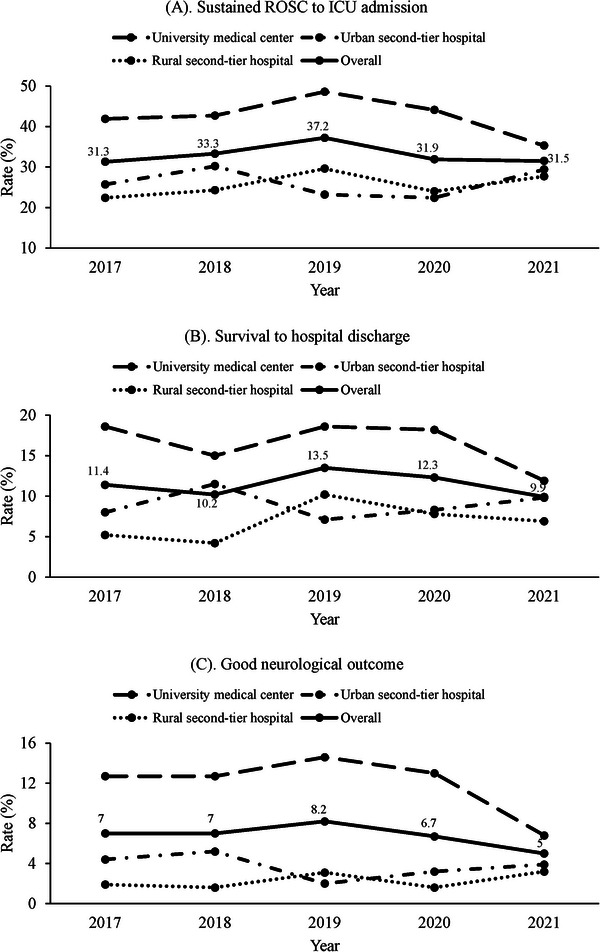
The outcomes of OHCA patients by years among the study hospitals: (A) sustained ROSC to admission; (B) survival to hospital discharge; (C) good neurological outcome. OHCA, out‐of‐hospital cardiac arrest; ROSC, return of spontaneous circulation.

Figure 2 further illustrates the year‐by‐year changes in outcomes from 2017 to 2021, stratified by hospitals. The detailed statistical comparisons of OHCA outcomes across the 3 hospitals for each year in this period (2017–2021) are included in the Supporting Information (Table S4). In sustained ROSC to admission, the university medical center displayed the highest rate in the initial year of the study (2017), exceeding 40%. However, a downward trend began in 2019. This trend was not seen in the other second‐tier hospitals. Additionally, in the years 2017, 2019, and 2020, the rate of sustained ROSC to admission was significantly lower in urban and rural second‐tier hospitals compared to the university medical center. In 2017, the urban second‐tier hospital had a rate of 25.7% compared to 41.9% in the university medical center, and the rural second‐tier hospital had a rate of 22.4% compared to 41.9% in the university medical center (*p* < 0.05). In 2019, the urban second‐tier hospital had a rate of 23.2% compared to 48.6% in the university medical center, and the rural second‐tier hospital had a rate of 29.6% compared to 48.6% in the university medical center (*p* < 0.05). In 2020, the urban second‐tier hospital had a rate of 22.4% compared to 44.1% in the university medical center, and the rural second‐tier hospital had a rate of 24.0% compared to 44.1% in the university medical center (*p* < 0.05).

Regarding the rate of survival to hospital discharge, there was a significant difference during the years 2017, 2019, and 2020. In 2017, the rate in the urban second‐tier hospital was 8.0% compared to 18.6% in the university medical center, and in the rural second‐tier hospital, it was 5.2% compared to 18.6% in the university medical center (*p* < 0.05). Similarly, in 2019, the rate in the urban second‐tier hospital was 7.1% compared to 18.6% in the university medical center, and in the rural second‐tier hospital, it was 10.2% compared to 18.6% in the university medical center (*p* < 0.05). In 2020, the rate in the urban second‐tier hospital was 8.3% compared to 18.2% in the university medical center, and in the rural second‐tier hospital, it was 7.8% compared to 18.2% in the university medical center (*p* < 0.05). Similarly, with the rate of good neurological outcome, the urban and the rural second‐tier hospitals in the years 2017, 2019, and 2020 were significantly lower than that of the university medical center (Table S4).

### 3.5 The association between variables and outcomes in the regression model

Table 3 shows the multivariable logistic regression model in analyzing the association between independent variables and outcomes. As compared with patients in the university medical center, patients in the urban second‐tier hospital had higher risk for poor outcomes (aOR, 0.67; 95% CI, 0.52–0.85 in sustained ROSC to ICU admission; aOR, 0.50; 95% CI, 0.30–0.84 in good neurological outcome). Patients in the rural second‐tier hospital had a higher risk for poor outcomes (aOR, 0.52; 95% CI, 0.42–0.65 in sustained ROSC to ICU admission; aOR, 0.46; 95% CI, 0.33–0.65 in survival to hospital discharge; aOR, 0.20; 95% CI, 0.12–0.34 in good neurological outcome). Patients who had prehospital ROSC or any shockable cardiac rhythm had higher odds ratio for good outcomes. On the other hand, patients who suffered OHCA during the COVID‐19 pandemic years did not have higher risk for poor outcomes.

**TABLE 3:** The association between intendent variables and outcomes in the multivariable logistic regression model.

Variables	Sustained ROSC to ICU admission		Survival to hospital discharge		Good neurological outcome	
	aOR (95% CI)	*P*	aOR (95% CI)	*P*	aOR (95% CI)	*P*
Age group, years						
30–39	Reference	–	Reference	–	Reference	–
40–49	1.93 (1.11–3.36)	0.020	1.39 (0.71–2.73)	0.343	1.10 (0.50–2.41)	0.813
50–59	1.96 (1.18–3.26)	0.009	1.49 (0.80–2.76)	0.207	0.96 (0.47–1.97)	0.905
60–69	1.35 (0.83–2.20)	0.233	0.98 (0.54–1.80)	0.954	0.73 (0.36–1.47)	0.376
Above 70	0.83 (0.52–1.33)	0.431	0.38 (0.21–0.69)	0.001	0.22 (0.11–0.44)	<0.001
Sex (male)	1.17 (0.97–1.41)	0.109	0.95 (0.72–1.27)	0.749	1.42 (0.95–2.12)	0.090
Hospital						
University medical center	Reference	–	Reference	–	Reference	–
Urban second‐tier hospital	0.67 (0.52–0.85)	0.001	0.82 (0.57–1.18)	0.288	0.50 (0.30–0.84)	0.008
Rural second‐tier hospital	0.52 (0.42–0.65)	<0.001	0.46 (0.33–0.65)	<0.001	0.20 (0.12–0.34)	<0.001
COVID‐19 pandemic years^a^	1.05 (0.84–1.31)	0.686	1.21 (0.88–1.66)	0.247	1.04 (0.69–1.58)	0.847
Prehospital ROSC	28.7 (18.1–45.3)	<0.001	14.0 (9.6–20.6)	<0.001	15.0 (9.4–23.8)	<0.001
Outdoor‐CPR	0.59 (0.36–0.96)	0.032	0.77 (0.38–1.55)	0.461	0.43 (0.15–1.20)	0.105
Airway management						
BVM	Reference	–	Reference	–	Reference	–
Direct laryngoscopy	3.08 (2.41–3.94)	<0.001‐	1.44 (1.01–2.06)	0.045	1.08 (0.66–1.76)	0.765
Video‐assisted	2.17 (1.63–2.89)	<0.001	0.77 (0.50–1.18)	0.222	0.51 (0.28–0.93)	0.029
Any shockable rhythm	2.36 (1.84–3.02)	<0.001	2.40 (1.70–3.37)	<0.001	4.28 (2.75–6.65)	<0.001

*Note*: Data were presented as adjusted odds ratio (95% confidence interval).

Abbreviations: aOR, adjusted odds ratio; CI, confidence interval; BVM, bag‐valve‐mask; CPR, cardiopulmonary resuscitation; ROSC, return of spontaneous circulation.

^a^ COVID‐19 pandemic years (2020 and 2021), non‐pandemic years (2017–2019) as a reference group.

## 4 LIMITATIONS

Some limitations may restrict the generalizability. First, the patients were recruited from only 1 university medical center and its affiliated hospitals. Although the sample size was large enough and the hospitals were geographically balanced (north, middle, and south of the country), selection bias may still result from a single‐center study. Second, information collection during the pandemic period about OHCAs from caregiver or medical records may have been more challenging than in the non‐pandemic period. Whether the COVID‐19 pandemic was associated with a specific OHCA population remains unknown. Finally, we could not follow the OHCA patients who were not brought to hospital but died at home. The number of documented OHCA patients was fewer than the actual OHCA patients. Further pooled results with a population‐based cohort study are warranted.

## 5 DISCUSSION

In this study, we conducted a comprehensive comparison of characteristics and outcomes in patients experiencing OHCA between the non‐pandemic period and the pandemic period. We also examined the differences in outcomes within each year, stratified by three different levels of hospitals. Our findings indicate that the outcomes in hospitals of different levels changed during the COVID‐19 pandemic. During the non‐pandemic period, the university medical center showed significantly better outcomes for OHCA compared to urban and rural second‐tier hospitals. However, during the pandemic period, these advantages were no longer seen.

We found that the OHCA outcomes during the pandemic period in the university medical center were poorer than those during the non‐pandemic period. First, it may result from a decrease in rescuers or limited capacity for resuscitation. During the pandemic, resuscitation team members were separated into 2 groups to avoid contamination. They discussed or organized resuscitation using wireless communication devices. Limited teamwork in the CPR‐designated room decreased resuscitation efficiency when compared with the standard working environment for emergency room CPR.^25^ This change may affect the university medical center mostly rather than the other 2 second‐tier hospitals. Second, the COVID‐19 pandemic may cause a decline in attendance to patients with ST‐elevation myocardial infarctions and cerebrovascular accidents, leading to worsening outcomes.^26^, ^27^ OHCA patients with potential acute coronary diseases may receive delayed treatment. The criteria of performing targeted temperature management also may have altered in response to COVID‐19 infection control. Third, limited resources and response times have unified resuscitation processes among hospitals, which may explain the consistent outcomes among hospitals.

In the meta‐analyses conducted by Scquizzato et al,^5^, ^6^ the COVID‐19 pandemic lowered the rate of survival, ROSC, and good neurological outcomes when compared with those in pre‐pandemic periods. TOR may confound these inconsistent results.^28^, ^29^ In fact, we did not know whether CPR was more easily terminated for OHCA patients with initial non‐shockable cardiac rhythms or multiple systematic diseases, and we do not have this data for each hospital. Some previous reports have indicated an increase in non‐shockable presenting cardiac rhythms during the COVID‐19 pandemic,^8^, ^10^, ^30^ and these results were pooled in a meta‐analysis.^6^ Our results inversely indicated that in 2020 and 2021, there was a decrease in immediate withdrawal rate of CPR in the ED and a significant decrease in the rate of termination of ongoing CPR when compared with the pre‐pandemic years (Figure S2). This decrease of announcing DNR was notably significant in the university medical center (approximately 50% decrease). To eliminate the potential effects confounded by incomplete resuscitation, we analyzed the subgroup for which ED physicians completed CPR without terminating the resuscitation process. We found that the rate of sustained ROSC to admission decreased, as well the rate of survival to hospital discharge, and the rate of good neurological outcomes, and no significant differences were found among hospitals during the pandemic period (Figure S3). These results were consistent with the pooled data in Scquizzato's study.^6^

In this study, we found an increase of video‐assisted intubation (Table S1). Because of consideration for high‐infective COVID‐19, rescuers should not get close to the patient's mouth when performing endotracheal tube intubation. The use of video‐assisted intubation increased. Direct laryngoscopy intubation was rarely used in the university medical center but was replaced by video‐assisted devices. However, there were still 2 of OHCA patients receiving direct laryngoscopy intubation in the second‐tier hospital. A sufficient and widespread supply of video‐assisted devices may be common in the university medical center as compared to the second‐tier hospital during the COVID‐19 pandemic. Additionally, the number of team members during resuscitation might influence the outcomes. Insufficient resuscitation rescuers made the outcomes worse in the outdoor‐CPR group as compared to the ED‐CPR group (Table S3).

Although there were no overall significant differences in OHCA outcomes between the non‐pandemic and pandemic period, the differences were found when stratified by hospital types. Specifically, the extent of the decrease in the rate of sustained ROSC to admission, survival to hospital discharge, and good neurological outcome varied among hospitals.

## AUTHOR CONTRIBUTIONS

All authors contributed substantially to this work. Chih‐Wei Sung and Edward Pei‐Chuan Huang originally conceived of the project. Cheng‐Yi Fan, Ching‐Yu Chen, Yun‐Chang Chen, Chi‐Hsin Chen, and Jiun‐Wei Chen initially collected data. Likwang Chen and Ching‐Yu Chen performed data analysis. Chih‐Wei Sung, Likwang Chen, and Wen‐Chu Chiang interpreted the results. Cheng‐Yi Fan wrote the manuscript. Edward Pei‐Chuan Huang, Chih‐Wei Sung, Wen‐Chu Chiang and Chien‐Hua Huang gave critical revisions. Edward Pei‐Chuan Huang took responsibility for the paper as a whole.

## CONFLICT OF INTEREST STATEMENT

The authors have no conflicts of interest to declare.

## Supporting information

Supporting Information

Supporting Information
