# Supplementary material for: Updated trends in the outcomes of out‐of‐hospital cardiac arrest from 2017–2021: Prior to and during the coronavirus disease (COVID‐19) pandemic
Source: J Am Coll Emerg Physicians Open. 2023 Nov 27;4(6):e13070. doi: 10.1002/emp2.13070 (PMC10680430; doi:10.1002/emp2.13070)
Supplement: Supplementary file 1 — Supporting Information [file EMP2-4-e13070-s001.docx]

| Table S1. Comparisons of the three study hospitals and EMS systems | | | |
| --- | --- | --- | --- |
| Hospital system | | | |
| Hospital size | University medical center | Urban second-tier hospital | Rural second-tier hospital |
| Hospital name | National Taiwan University Hospital | National Taiwan University Hospital Hsin-Chu Branch | National Taiwan University Hospital Yun-Lin Branch |
| Location | Taipei City | Hsin-Chu City | Yun-Lin County |
| Beds | >2500 | 600-700 | 700-800 |
| ICU beds | 195 | 50 | 18 |
| ED visit per year | 100,000 | 55,000 | 45,000 |
| ECMO | Yes | yes | yes |
| TTM | Yes | yes | yes |
| EMS system | | | |
| Number of Ambulances | 92 | 64 | 39 |
| Crew configuration | 2 teams deployed; at least 1 EMT-P | 1 team deployed; EMT-P not always available | 1 team deployed; EMT-P not always available |
| Calls per year | 120,000 | 40,000 | 30,000 |
| BLS units | yes | yes | yes |
| Epinephrine administration | Performed by EMT-P | Performed by EMT-P | Performed by EMT-P |
| Supraglottic airway | yes | Yes | yes |
| Endotracheal tube intubation | Performed by EMT-P | Performed by EMT-P | Performed by EMT-P |
| Intraosseous access | Performed by EMT-P | Performed by EMT-P | Performed by EMT-P |
| BLS: basic life support; ED: emergency department; EMS: emergency medical service; EMT-P: emergency medical technician-paramedic ICU: intensive care unit | | | |

| **Table S2.** Out-of-hospital cardiac arrest characteristics, stratified by year | | | | | | |
| --- | --- | --- | --- | --- | --- | --- |
|  | 2017 | 2018 | 2019 | 2020 | 2021 | *p* |
| Characteristic | (n= 559) | (n= 498) | (n= 548) | (n= 595) | (n= 619) |  |
| Age | 71.5 ± 16.0 | 71.4 ± 16.0 | 71.1 ± 16.4 | 70.0 ± 16.1 | 71.0 ± 15.9 | 0.525 |
| Males | 339 (60.6) | 300 (60.2) | 330 (60.2) | 360 (60.5) | 387 (62.5) | 0.921 |
| BMI | 22.9 ± 5.1 | 22.6 ± 4.9 | 23.3 ± 4.9 | 23.5 ± 5.1 | 23.2 ± 5.1 | 0.121 |
| Hospital |  |  |  |  |  | 0.004 |
| University medical center | 236 (42.2) | 213 (42.8) | 253 (46.2) | 247 (41.5) | 278 (44.9) |  |
| Urban second-tier hospital | 113 (20.2) | 96 (19.3) | 99 (18.1) | 156 (26.2) | 153 (24.7) |  |
| Rural second-tier hospital | 210 (37.6) | 189 (38.0) | 196 (35.8) | 192 (32.3) | 188 (30.4) |  |
| Prehospital ROSC | 50 (8.9) | 43 (8.6) | 47 (8.6) | 48 (8.1) | 35 (5.7) | 0.211 |
| Outdoor-CPR | 0 (0.0) | 0 (0.0) | 0 (0.0) | 7 (1.2) | 141 (22.8) | <0.001 |
| Immediate DNR at ED | 81 (14.5) | 74 (14.9) | 70 (12.8) | 69 (11.6) | 63 (10.2) | 0.092 |
| TOR at ED | 138 (24.7) | 110 (22.1) | 114 (20.8) | 98 (16.5) | 101 (16.3) | 0.001 |
| Airway management |  |  |  |  |  |  |
| Direct laryngoscopy | 309 (55.3) | 275 (55.2) | 287 (52.4) | 147 (24.7) | 88 (14.2) | <0.001 |
| Video-assisted | 23 (4.1) | 36 (7.2) | 43 (7.8) | 264 (44.4) | 286 (46.2) | <0.001 |
| SGA | 0 (0.0) | 4 (0.8) | 5 (0.9) | 6 (1.0) | 46 (7.4) | <0.001 |
| Tracheostomy | 3 (0.5) | 2 (0.4) | 4 (0.7) | 10 (1.7) | 8 (1.3) | 0.137 |
| Any shockable rhythm | 65 (11.6) | 61 (12.2) | 60 (10.9) | 88 (14.8) | 87 (14.1) | 0.241 |
| CPR duration | 21.6 ± 16.0 | 19.9 ± 14.9 | 18.9 ± 14.5 | 22.2 ± 14.5 | 19.9 ± 13.5 | 0.005 |
| BMI: body mass index; CPR: cardiopulmonary resuscitation; DNR: do not resuscitate; ED: emergency department; ROSC: return of spontaneous circulation; SGA: supraglottic airway; TOR: termination of resuscitation | | | | | | |

| **Table S3.** Comparison in covariates and outcomes by resuscitation place | | | |
| --- | --- | --- | --- |
|  | ED-CPR | Outdoor-CPR | *p* |
| Characteristic | (n= 2,671) | (n= 148) |  |
| Age | 71.0 ± 16.0 | 71.1 ± 15.4 | 0.956 |
| Males | 1622 (60.7) | 94 (63.5) | 0.499 |
| BMI | 23.1 ± 5.0 | 23.0 ± 5.0 | 0.911 |
| Hospital |  |  | <0.001 |
| University medical center | 1086 (40.7) | 141 (95.3) |  |
| Urban second-tier hospital | 610 (22.8) | 7 (4.7) |  |
| Rural second-tier hospital | 975 (36.5) | 0 (0) |  |
| Prehospital ROSC | 217 (8.1) | 6 (4.1) | 0.074 |
| Immediate DNR at ED | 349 (13.1) | 8 (5.4) | 0.006 |
| TOR at ED | 545 (20.4) | 16 (10.8) | 0.004 |
| Airway management |  |  |  |
| Direct laryngoscopy | 1091 (40.8) | 15 (10.1) | <0.001 |
| Video-assisted | 600 (22.5) | 52 (35.1) | <0.001 |
| SGA | 27 (1.0) | 34 (23.0) | <0.001 |
| Tracheostomy | 26 (1.0) | 1 (0.7) | 1 |
| Any shockable rhythm | 345 (12.9) | 16 (10.8) | 0.456 |
| CPR duration | 20.6 ± 14.9 | 19.5 ± 11.0 | 0.400 |
| Sustained ROSC to admission | 892 (33.4) | 38 (25.7) | 0.052 |
| Survival to hospital discharge | 311 (11.6) | 12 (8.1) | 0.189 |
| Good neurological outcome | 185 (6.9) | 5 (3.4) | 0.094 |
| BMI: body mass index; CPR: cardiopulmonary resuscitation; DNR: do not resuscitate; ED: emergency department; ROSC: return of spontaneous circulation; SGA: supraglottic airway; TOR: termination of resuscitation | | | |

| **Table S4.** Statistical details on comparison of out-of-hospital cardiac arrest outcomes among three hospitals in each year (2017 - 2021) | | | |
| --- | --- | --- | --- |
| (A) Sustained ROSC to admission (%) | |  |  |
|  | University medical center | Urban second-tier hospital | Rural second-tier hospital |
| 2017 | 41.9 | 25.7* | 22.4* |
| 2018 | 42.7 | 30.2 | 24.3* |
| 2019 | 48.6 | 23.2* | 29.6* |
| 2020 | 44.1 | 22.4* | 24* |
| 2021 | 35.3 | 29.4 | 27.7 |
| (B) Survival to hospital discharge (%) | | | |
|  | University medical center | Urban second-tier hospital | Rural second-tier hospital |
| 2017 | 18.6 | 8* | 5.2* |
| 2018 | 15 | 11.5 | 4.2* |
| 2019 | 18.6 | 7.1* | 10.2* |
| 2020 | 18.2 | 8.3* | 7.8* |
| 2021 | 11.9 | 9.8 | 6.9 |
| (C) Good neurological outcome (%) | | | |
|  | University Medical center | Urban second-tier hospital | Rural second-tier hospital |
| 2017 | 12.7 | 4.4* | 1.9* |
| 2018 | 12.7 | 5.2 | 1.6* |
| 2019 | 14.6 | 2* | 3.1* |
| 2020 | 13 | 3.2* | 1.6* |
| 2021 | 6.8 | 3.9 | 3.2 |
| The symbol “*” indicates that the value in this year significantly differs from that in the university medical center (*p*<0.05) | | | |

ROSC: return of spontaneous circulation;
