# Supplementary material for: Updated trends in the outcomes of out‐of‐hospital cardiac arrest from 2017–2021: Prior to and during the coronavirus disease (COVID‐19) pandemic
Source: J Am Coll Emerg Physicians Open. 2023 Nov 27;4(6):e13070. doi: 10.1002/emp2.13070 (PMC10680430; doi:10.1002/emp2.13070)
Supplement: Supplementary file 2 — Supporting Information [file EMP2-4-e13070-s002.docx]

**Supplementary figure legends**

Figure S1. OHCA etiology from 2017 through 2021


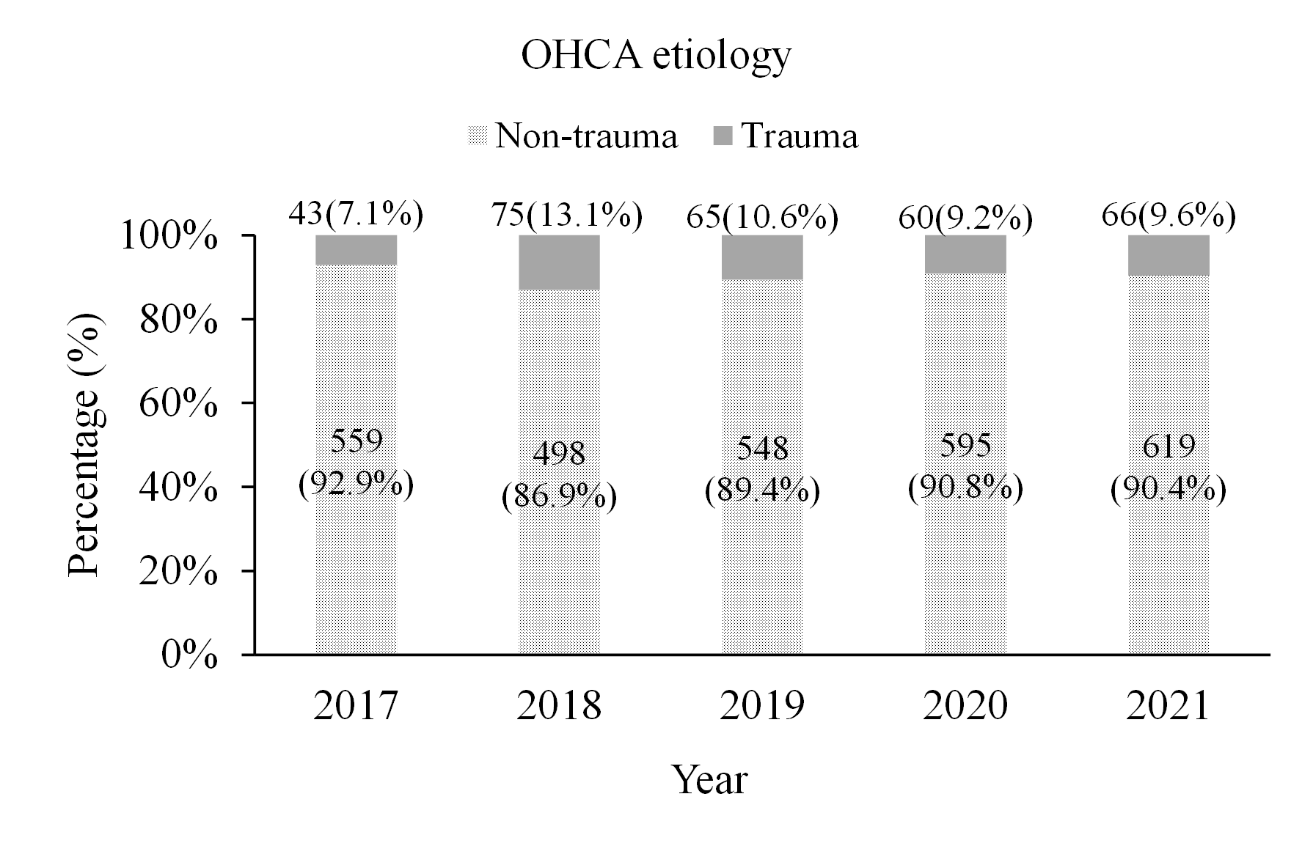


Figure S2. The trend of “do not resuscitate” from 2017 through 2021 in three hospitals.





Figure S3. The OHCA outcomes in the subgroup who did not withdraw or terminate resuscitation, among the study hospitals: (A) sustained ROSC to admission; (B) survival to hospital discharge; (C) good neurological outcome.
